# A review of the occurrence and diversity of the sphragis in butterflies (Lepidoptera, Papilionoidea)

**DOI:** 10.3897/zookeys.694.13097

**Published:** 2017-08-29

**Authors:** Ana Paula S. Carvalho, Albert G. Orr, Akito Y. Kawahara

**Affiliations:** 1 Entomology and Nematology Department, University of Florida, 1881 Natural Area Dr, Gainesville, FL 32608, United States; 2 McGuire Center for Lepidoptera and Biodiversity, Florida Museum of Natural History, University of Florida, 3215 Hull Road, Gainesville, FL, 32611 United States; 3 Environmental Futures Research Institute, Griffith University, Nathan, QLD 4111, Australia

**Keywords:** Ditrysia, mate conflict, mating plug, Nymphalidae, Papilionidae, sperm guarding, sperm precedence, sexual competition

## Abstract

Males of many butterfly species secrete long-lasting mating plugs to prevent their mates from copulating with other males, thus ensuring their sperm will fertilize all future eggs laid. Certain species have further developed a greatly enlarged, often spectacular, externalized plug, termed a sphragis. This distinctive structure results from complex adaptations in both male and female genitalia and is qualitatively distinct from the amorphous, internal mating plugs of other species. Intermediate conditions between internal plug and external sphragis are rare. The term sphragis has often been misunderstood in recent years, hence we provide a formal definition based on accepted usage throughout most of the last century. Despite it being a highly apparent trait, neither the incidence nor diversity of the sphragis has been systematically documented. We record a sphragis or related structure in 273 butterfly species, representing 72 species of Papilionidae in 13 genera, and 201 species of Nymphalidae in 9 genera. These figures represent respectively, 13% of Papilionidae, 3% of Nymphalidae, and 1% of known butterfly species. A well-formed sphragis evolved independently in at least five butterfly subfamilies, with a rudimentary structure also occurring in an additional subfamily. The sphragis is probably the plesiomorphic condition in groups such as Parnassius (Papilionidae: Parnassiinae) and many Acraeini (Nymphalidae: Heliconiinae). Some butterflies, such as those belonging to the *Parnassius simo* group, have apparently lost the structure secondarily. The material cost of producing the sphragis is considerable. It is typically offset by production of a smaller spermatophore, thus reducing the amount of male-derived nutrients donated to the female during mating for use in oogenesis and/or somatic maintenance. The sphragis potentially represents one of the clearest examples of mate conflict known. Investigating its biology should yield testable hypotheses to further our understanding of the selective processes at play in an ‘arms race’ between the sexes. This paper provides an overview, which will inform future study.

## Introduction

Male butterflies, like most animals, typically maximize their reproductive success by mating with as many females as possible. Conversely, for females, one copulation is normally sufficient to provide sperm to fertilize all the eggs that they can produce. Female butterflies possess sperm storage organs which can maintain vital sperm from a single mating for their entire life (Chapman 1969, Parker 1970, 1984, Ferro and Akre 1975, Ehrlich and Ehrlich 1978, Walker 1980, Thornhill and Alcock 1983, Drummond 1984, Gwynne 1984, Orr 1988, Solensky and Oberhauser 2009, Klowden 2013). However, females frequently mate more than once. This may result from the need for: more male-derived nutrients received during copulation, more sperm (especially if their first mate was depleted), increasing the genetic quality of the fertilized eggs by mating with a fitter male, increased genetic diversity of offspring in an unpredictable environment, or reducing the energy loss and risk of harm involved in resisting the attempts of copulation with new males. With many factors potentially influencing female mating, there is great interspecific variation in female mating frequencies, ranging from obligate monandry to regular polyandry (Scott 1972, Walker 1980, Thornhill and Alcock 1983, Knowlton and Greenwell 1984, Arnqvist and Nilsson 2000, Blanckenhorn et al. 2002, Wedell 2005).

When female insects mate more than once, it is often the sperm of the final male to mate which fertilizes most (or all) of the female’s remaining eggs, a process termed “last male sperm precedence” (Labine 1966, Parker 1970, Boggs and Watt 1981, Simmons 2001). Males of all Lepidoptera, except Micropterigidae (Sonnenschein and Hauser 1990), produce infertile (apyrene) sperm, which may play a role in blocking those of a previous partner (Silberglied et al. 1984). Therefore, when a male mates, it is generally in his interest to prevent the female from mating again. Conversely, if the female benefits from polyandry, counter-mechanisms to overcome male paternity assurance strategies may evolve.

Although many strategies are found among insects to prevent females from remating, with females evidently complicit in some cases (Parker 1970, 1984, Gilbert 1976, Thornhill and Alcock 1983, T. Chapman and Patridge 1996, Orr 1999a, Simmons 2001, Hosken et al. 2009, Shuker and Simmons 2014), in butterflies the most common strategy is for the male to produce a mating plug which physically blocks the copulatory opening (Labine 1966, Parker 1970, Ehrlich and Ehrlich 1978, Drummond 1984, Orr and Rutowski 1991, Matsumoto and Suzuki 1995, Orr 1995). Mating plugs also occur in mammals, nematodes and in many arthropod groups, such as spiders, wasps, and flies (Gillies 1956, Thornhill and Alcock 1983, Timmermeyer et al. 2010, Uhl et al. 2010, Hirt et al. 2017). However, only in those of the ditrysian Lepidoptera, a group to which all butterflies belong, can the plug be potentially permanent because it does not impede oviposition. This stems from the unique arrangement of the female genital ducts in the Ditrysia. The copulatory opening (ostium bursae) is located ventrally on the eighth segment, typically within a shallow pocket, the sinus vaginalis, and is completely separate from the oopore, which exits terminally between the paired ovipositor lobes (papillae anales) and through which eggs pass during oviposition (Eaton 1988, Scoble 1992). Therefore, not only can the plug be permanent, it can also be large and externally elaborate, covering large parts of the female’s abdomen, in which case it is termed a sphragis.

### The history and nature of the sphragis

The production of a sphragis, one of the more extreme male strategies that has evolved to prevent the female from remating, occurs only in certain butterflies. It was first clearly described by Linnaeus (1746) for the parnassiine swallowtail *Parnassius apollo* (Linnaeus 1758) and was subsequently discussed in more than 140 publications before 1918 (Bingham 1907, Houlbert 1916, Bryk 1919). The term ‘sphragis’ (plural: *sphragides*), which means ‘seal’ in Greek, was first used by Eltringham (1912), who described the structure found in many *Acraea* Fabricius, 1807 species (Nymphalidae: Heliconiinae) as a “*wax-like seal [produced] after pairing*”. The term was also used by Waterhouse and Lyell (1914) for the postcopulatory ‘seal’ in *Cressida cressida* (Fabricius, 1775) (Papilionidae: Troidini) and by Bryk (1919), who applied the term to the structure in most genera where a sphragis is recognized as occurring today. With this level of sustained attention over the decades, it is clear that the sphragis is a recognizable phenomenon deserving special investigation. However, there has been recent confusion in the use of the term, hence there is a need to clarify its meaning. We therefore define the sphragis as: an external formation, originating from male accessory glands, with a well-defined, species-specific structure, fixed to the female abdomen following insemination, where it blocks the ostium bursae.

The form of the sphragis is consistent within a species, it is shaped by complex adaptations in the male genitalia, and it is wholly or mainly external to the female abdomen. In these respects, sphragides differ qualitatively from smaller amorphous internal mating plugs. In almost all sphragis-bearing species, the female external genitalia are strongly modified from the ditrysian groundplan; these modifications have in turn influenced the form of the sphragis (Orr 1988, 1995). Intermediate states, where they occur, involve highly sophisticated male and/or female adaptations (Orr 1988).

Specialized features of sphragides in different species have been identified by several authors (Bryk 1918, Ackery 1975, Pierre 1985a, Orr 1988, 1999a, Matsumoto and Suzuki 1995). In some, the sphragis incorporates long scales derived from specialized tufts on the male genitalia (Pierre 1985a, Orr and Kitching 2010, Matsumoto, Orr & Yago *in prep*.). These may reinforce the structure of the sphragis and/or provide bulk by increasing the sphragis in size and weight. In others, the sphragis is densely tiled with short flat scales which may make it slippery and difficult for other males to grasp. In both cases, the scales seem to hinder sphragis removal by other males (Pierre 1985a, Orr 1999b). The sphragis is also frequently hollow, greatly increasing its bulk, or is intricately sculptured, often including projections which potentially make access to the female genitalia difficult. Moreover, an elaborate girdle, which encircles the female abdomen, is often associated with the sphragis, holding it tightly in place. It sometimes incorporates strengthening scales.

One oft-cited misconception is that the ‘waxy’ sphragis can dissolve in water (Drummond 1984, Epstein 1987, Sourakov and Emmel 1997), supposedly explaining the high incidence of sphragis-bearing species in semiarid habitats. This fallacy originated from a misreading by Hinton (1964) of the French abstract of Tykac (1951), which was originally published in Czech (Orr 1988).

### Formation of the sphragis

The sphragis is produced from a viscous secretion which is molded within membranous or sclerotized pockets in the male genitalia, sometimes being extruded gradually as it hardens, so that the final product is far larger than any cavity in the male’s body (Bryk 1918, Takakura 1967, Matsumoto 1987, Orr 1988, 1995). Hardening may occur on contact with air, but this process is not understood. It is also possible that sphragis formation is mediated by enzymatic action, at least in some species (Orr 1995). The sphragidal fluid varies among species, with some producing an almost clear vitreous secretion, and others a secretion with an appearance similar to the lipoprotein mass found in a fresh spermatophore (Orr 1995). The precise composition of the sphragis has not been established, but it is known to contain high protein levels (Orr 1988), and its waxy appearance suggests a lipid element. All sphragis-bearing species that have been investigated exhibit hypertrophied, paired accessory glands and it is generally accepted that these secrete the sphragidal fluid (Eltringham 1925, Ehrlich 1961, Orr 1988, 1995, 2002). It is known that the spermatophore, which in most butterflies is a thick hollow structure formed from lipoprotein (Boggs and Gilbert 1979, Orr 1988), is secreted by a glandular region of the ductus ejaculatorius simplex (Callahan and Cascio 1963), or ejaculatory duct, which leads directly to the aedeagus.

Production of the sphragis is a substantial male material investment, which apparently occurs at the expense of the spermatophore. A great deal of material needs to be repurposed to build the sphragis. The amount of accessory gland secretion (including the spermatophore, and spermatophylax), which females could potentially metabolize and utilize for oogenesis or somatic maintenance (Boggs and Gilbert 1979, Boggs and Watt 1981, Orr 1988, 1995), is thus reduced in its subsequent transfer to the female. Even while producing a small spermatophore, males are limited in the number of sphragides they can produce (Orr 1988, 1995). It is common to find specimens (both in the field and in museum collections) with frail or incomplete sphragides. These were probably produced by males that recently mated or had mated several times previously, and thus had exhausted the resources necessary to produce a sphragis of normal bulk (Orr 1988). It has been estimated that males of the *Heteronympha penelope* Waterhouse, 1937 (Nymphalidae: Satyrinae) require 7–10 days after mating to recover the resources necessary to produce another sphragis, and can only produce 3–4 effective sphragides in their lifetime (Orr 2002). Available measurements indicate that the investment involved in the production of each sphragis varies between about 3–20% of the male’s body weight, depending on the species (Table 1) (Orr 1988).

**Table 1.** Percentage of male investment in the sphragis based on male’s weight. Data adapted from Orr (1988).

	Male investiment on the sphragis (% body weight)
*Parnassius glacialis* (Papilionidae: Parnassiinae)	20.5 ± 2.7
*Parnassius apollo* (Papilionidae: Parnassiinae)	7.4 ± 2.5
*Luehdorfia japonica* (Papilionidae: Parnassiinae)	9.8 ± 5.7
*Cressida cressida* (Papilionidae: Papilioninae)	6.7 ± 1.1
*Euryades corethrus* (Papilionidae: Papilioninae)	8.6 ± 1.0
*Parides proneus* (Papilionidae: Papilioninae)	3.1 ± 1.5
*Acraea serena* (Nymphalidae: Heliconiinae)	3.3 ± 0.4
*Acraea anemosa* (Nymphalidae: Heliconiinae)	6.1 ± 0.9
*Acraea andromacha* (Nymphalidae: Heliconiinae)	3.1 ± 0.2

### Behavioral specialization

Presence of the sphragis is typically associated with specific behavioral patterns. Courtship behavior is absent or rudimentary in almost all sphragis-bearing species (Epstein 1987, Matsumoto 1987, Orr 1988, Tyler et al. 1994, Sourakov and Emmel 1997). Mates are secured, often by pursuit and aerial capture, with mating taking place in mid-air or on the ground (Larsen 1991, 2005). Matsumoto (1987) reported that males of *Luehdorfia japonica* Leech, 1889 (Parnassiinae) seize females in midair, carry them to the ground, and copulate with them. Similar behavior has been analyzed in detail in the Australian sphragis-bearing troidine *C. cressida* (Orr 1988, 1999b). In this species, large males often capture and mate with the smaller females in mid-air but relatively smaller males carry females to the ground to mate. *Cressida cressida* males are significantly larger than females, an unusual condition in butterflies, but this disparity probably relates to their strong territorial behavior, rather than to success at forced copulation (Orr 1999b).

### Function of the sphragis

All experimental evidence (Matsumoto 1987, Orr 1988), indicates that the sphragis functions as a physical barrier, preventing a new male from penetrating the female’s ostium bursae and reinseminating her. In his monumental work, *Grundzüge der Sphragidologie*, Bryk (1918, 1919, 1930, 1950) effectively dismissed much fanciful speculation that had taken place in the preceding 170 years, arguing that the sphragis serves as a physical barrier to insemination. This conclusion was reached earlier by Marshall (1901, 1902), based on his observations of *Acraea* (Heliconiinae) mating in nature, and Haude (1913), based on observations of *Parnassius*. Eltringham (1925) reached this conclusion, noting at the time ‘*indeed it would seem that more has been written about [the sphragis], and with less result, than about most features of insect structure*’. Because the sphragis blocks only the genital opening of the female, the ovipositor is unobstructed (Labine 1964), although the sphragis can be so large in many species it can be a distinct encumbrance and accidental blocking of the oopore has been reported (van der Poorten and van der Poorten 2016). The size and shape of the sphragis could potentially be important in preventing males from being able to grasp the female with their valvae (Orr 1995). Moreover, Orr and Rutowski (1991) showed that the sphragis of *C. cressida* might have the secondary function of providing a visual cue to males indicating that a female has already mated. In their study, females with intact sphragides were less likely to be pursued by males when compared with females where the sphragis was experimentally trimmed or removed. Supporting this hypothesis is the observation that females of *C. cressida* have evolved a specialized posture, that when in flight, allows the sphragis to be seen clearly from behind (Orr and Rutowski 1991, Orr 1999b). It is conceivable that in other sphragis-bearing species, males might also be able to visually assess whether mating is possible depending on the development of the sphragis (Orr and Rutowski 1991). Apart from its large size, the sphragis often stands out with strong color contrast to the rest of the body, possibly increasing its apparency, but if this occurs it is surely a secondary function (Orr 1999b).

### The taxonomic distribution of the sphragis

In the most comprehensive comparative survey to date, Orr (1988) examined the presence or absence of mating plugs, the relative investment in spermatophore versus plug, as well as female genital anatomy in over 160 species, including sphragis-bearing species and others representing a range of plugging strategies in the Papilionidae, Pieridae and Nymphalidae. Bryk (1919, 1935b) surveyed the sphragis of several Papilionidae and Palaearctic Nymphalidae genera, while Ackery (1975) focused on Parnassiinae. Although most sphragis-bearing genera recognized by contemporary taxonomic arrangements were included, no study so far has provided a comprehensive survey of all species. The morphology of the sphragis and associated male and female genitalia are well documented in widely scattered literature for African *Acraea* (Eltringham 1912, 1925, van Son 1963, Pierre 1985a, 1988, 1992a, 1992b, Orr 1988), but the sphragis in Neotropical *Acraea* (Paluch et al. 2003) has received less attention.

### The present state of knowledge

Studies on the biology of the sphragis in living butterflies have mostly focused on *C. cressida* in the Troidini (Orr 1988), *Luehdorfia* and *Parnassius* in the Parnassiinae (Matsumoto 1987), and *H. penelope* in the Satyrinae (Orr 2002). Some less detailed observations are also available for the genus *Acraea* in the Heliconiinae (Marshall 1901, Eltringham 1912, Epstein 1987, Orr 1988).

A large body of published information exists on the occurrence and diversity of the sphragis, but it is largely obscure, scattered, old and often published in languages other than English. There is a need for this information to be collated and for several conspicuous gaps to be filled. In this paper we aim to provide an overview of the subject and develop a dataset to inform future investigation. We present the first comprehensive review of the structural variability of the sphragis across all butterflies, illustrating the variety of forms that these can take. All species of butterflies in which a well-defined sphragis is known to occur are listed based on published information and direct observation of museum specimens. Reports of sphragis occurrence in Erebidae (Rawlins 1992) and Lycaenidae (Bryk 1918, 1919) we dismiss as erroneous.

Bryk (1919) illustrated a sphragis-like structure in the Nymphalidae *Argynnis paphia*, however, subsequent investigation has failed to confirm this observation and it is suggested (Matsumoto pers. comm.) that the structure observed in this case is a result of an accumulation of several mating plugs deposited by one or more males after multiple matings by the female, which could be externally observed.

In addition, as noted previously, sphragis-like formations which may represent incipient sphragis evolution or secondary loss occur in some butterflies (Orr 1995). A complex anomalous structure is also discussed in our analysis. We establish a three point system of categorization for true sphragides based on degree of complexity. Finally, we examine the processes and patterns of sphragis evolution in the context of a currently accepted butterfly phylogeny.

## Methods

We examined butterfly specimens in museum collections and searched historical and recent literature for reports, descriptions and illustrations of sphragides or similar external structures (Guenée 1872, Eltringham 1912, Bryk 1935a, Riley 1939, van Son 1963, Saigusa 1973, Ackery 1975, Common and Waterhouse 1981, Saigusa and Lee 1982, Parsons 1983, D’Abrera 1984, 1990, 1995, 1997, Drummond 1984, Pierre 1985a, 1985b, Matsumoto 1987, Miller 1987, Orr 1988, Matsumoto and Suzuki 1995, Penz and Francini 1996, Francini et al. 2004, Paluch et al. 2006, Churkin 2006, Neild 2008, Neild and Romero 2008, Pierre and Bernaud 2009, 2013, Bollino and Racheli 2012, Harada et al. 2012). Museum specimens were studied by APSC and AGO. The former visited the Cornell University Insect Collection, Ithaca, NY, USA (CUIC), Florida Museum of Natural History, McGuire Center for Lepidoptera and Biodiversity, Gainesville, FL, USA (MGCL), and the National Museum of Natural History, Washington DC, USA (USNM). AGO studied specimens in the Australian National Insect Collection, Canberra, Australia, (ANIC), the Natural History Museum, London, UK (BMNH), the Museum Alexander Koenig, Bonn, Germany (ZFMK), and the private collection of the late Prof. Dr. Clas Naumann (Bonn). For our museum specimen searches, we especially targeted Nymphalidae and Papilionidae because only in these families has the sphragis been reliably reported by previous authors. Mated females of all species available to us in each target group (see below) were inspected using a dissecting microscope with the following aims: (1) to confirm and categorize the sphragis in species previously reported to have a sphragis, and (2) to gather new data for any species not mentioned in literature. We also contacted specialists who had studied sphragis-bearing species in order to gather additional unpublished data.

For each species, we tried to examine mated females of at least ten specimens if possible (in a few cases thousands were available). Generally, the minimum combined sample was five specimens. Overall, we directly examined approximately 80% of all sphragis bearing species. We recorded key traits of the sphragis of each species, especially: the presence of male scales attached to the surface or incorporated into its matrix; whether it was mainly hollow or solid; the presence of projections or other specialized sculpturing; and its size relative to the female abdomen. We also examined structures previously classified as protosphragides or vestigial sphragides, and we define an anomalous form as a ‘hemi-sphragis’. We targeted the following taxa based on published and unpublished information: Nymphalidae: *Acraea*, *Amauris* Hübner, 1816, *Argynnis* Fabricius 1807, *Dircenna* Doubleday, 1847, *Hestina* Westwood, 1850, *Heteronympha* Wallengren, 1858, *Hipparcha* Fabricius 1807, *Pteronymia* Butler & Druce, 1872, *Sasakia* Moore 1896; Papilionidae: Parnassiinae, *Cressida* Swainson, 1832, *Euryades* Felder & Felder, 1864, *Losaria* Moore, [1902], *Parides* Hübner, 1819, and *Trogonoptera* Rippon, [1890].

In order to show the extent of interspecific variation in sphragis morphology, we digitally imaged the sphragis of representative species using a Canon 5D MKIII camera body and a Canon MP-E 65mm lens. For each image, 10 to 20 image layers (depending on the size of the sphragis) were taken across a series of close-spaced focal planes, using the Automated Macro Rail for Focus Stacking StackShot. These were later stacked using the software Helicon Focus on a PC computer. Figures were edited and assembled using Adobe Photoshop CS4. The taxonomic classification in this study follows Häuser (2005) for Papilionidae, Pierre and Bernaud (2014a) for Acraeini, Lamas (2004) for the Neotropical non-Acraeini Nymphalidae, and LepIndex (Beccaloni et al. 2003) for the remaining Nymphalidae.

### Terms related to sphragis-like structures used in this study

• *Protosphragis*: amorphous, non-species-specific version of the sphragis, often facultative in the groups where it is found, and potentially associated with groups in the early evolution of the sphragis.

• *Vestigial sphragis*: non-species specific version of the sphragis, irregular in occurrence, associated with groups believed to be in the process of losing the sphragis.

• *Hemi-sphragis*: semi-internal version of the sphragis, with a complex and regular internal arrangement of lacunae used to increase bulk as well as a regularly striated exposed outer face, associated with complex adaptations in male genitalia, thus equivalent to true sphragides in terms of complexity and regularity of form. Restricted to the troidine genus *Trogonoptera* and figured in Orr (1995).

Based on the traits recorded for each sphragis we developed a system of categorization based on level of complexity. Category 1 (low complexity): a protosphragis or a vestigial sphragis, characterized by being small, amorphous and of facultative occurrence. Category 2 (moderate complexity): a hemi-sphragis, or a well-formed externalized sphragis lacking male scales and essentially solid, of small to medium size. Category 3 (medium complexity): a well formed sphragis incorporating male scales but solid and of simple form, mostly small to medium in size. Category 4 (high complexity): large to very large sphragides, hollow and/or with specialized projections, girdles, or other complex sculpturing, further defined based on absence (Category 4a) or presence (Category 4b) of scales.

## Results and discussion

**Figures 1–8.**
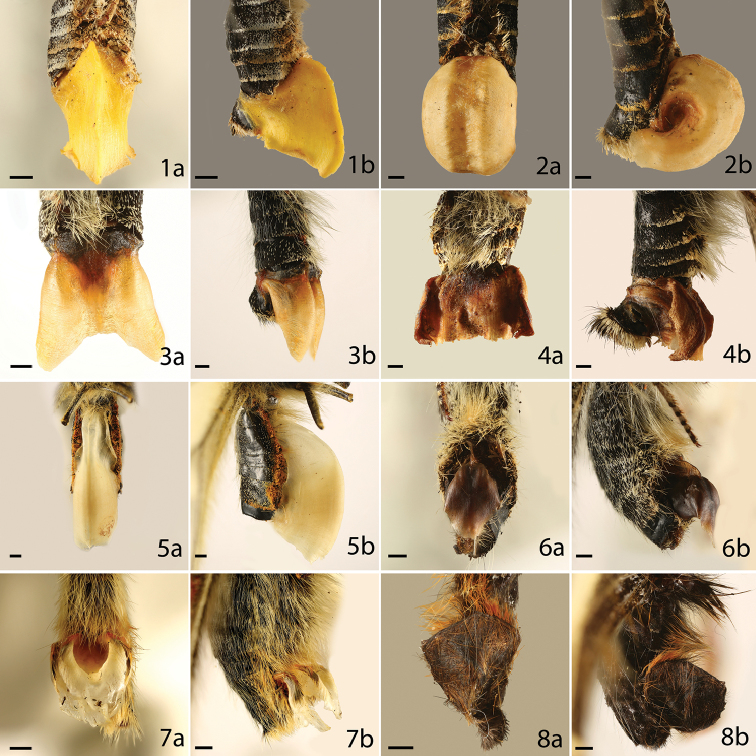
Sphragis of butterfly species **a** ventral **b** lateral, category of the sphragis in parenthesis. **1** *Parnassius autocrator* (4) **2** *P. charltonius* (4) **3** *P. delphius* (4) **4** *P. imperator* (4) **5** *P. mnemosyne* (4) **6** *P. phoebus* (4) **7** *P. tenedius* (4) **8** *Luehdorfia chinensis* (3). Scale bar = 1 mm.

**Figures 9–11.**
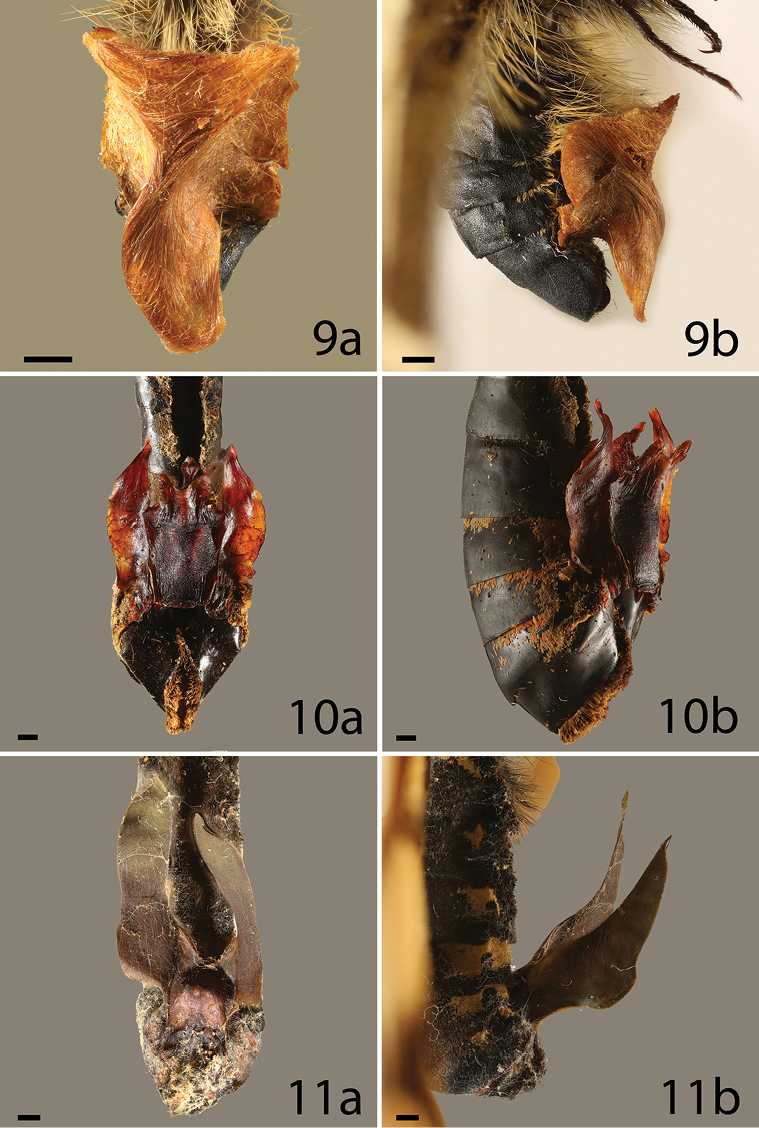
Sphragis of butterfly species, **a** ventral **b** lateral, category of the sphragis in parenthesis. **9** *L. puziloi* (4) **10** *Cressida cressida* (4) **11** *Euryades duponchelii* (4). Scale bar = 1 mm.

**Figures 12, 13.**
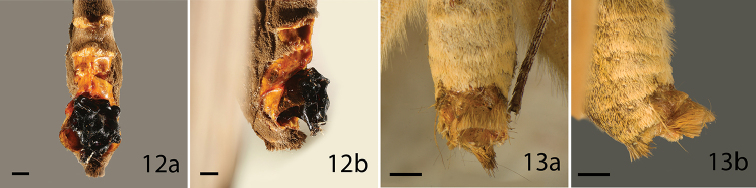
Sphragis of butterfly species, **a** ventral **b** lateral, category of the sphragis in parenthesis. **12** *Amauris niavius* (1) **13** *H. penelope* (4). Scale bar = 1 mm.

**Figures 14–23.**
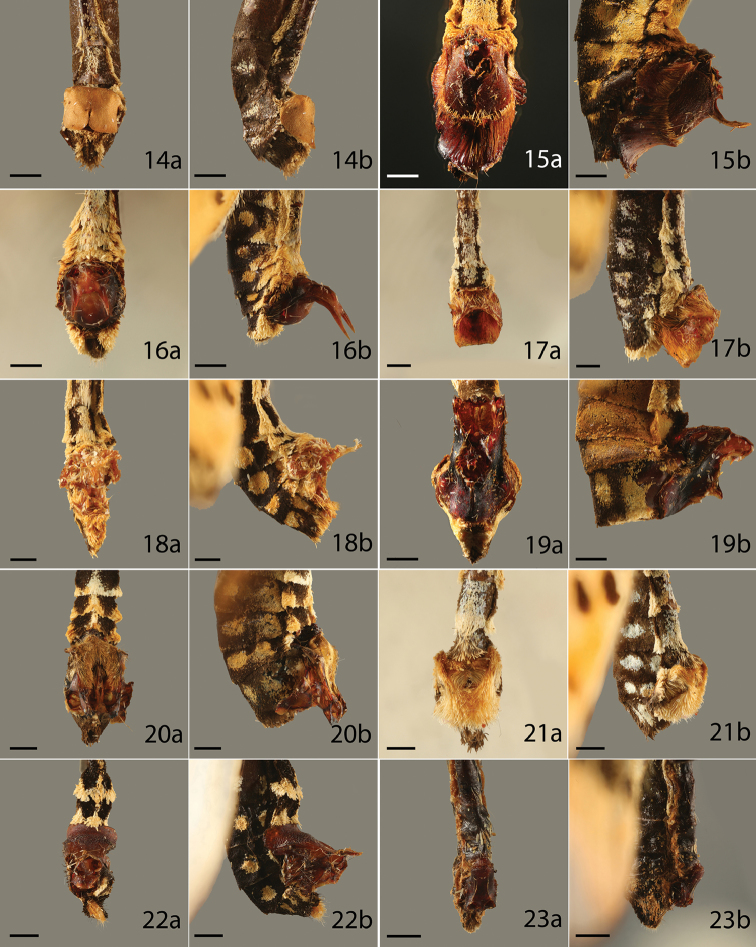
Sphragis of butterfly species, **a** ventral, **b** lateral, category of the sphragis in parenthesis. **14** *Acraea kraka* (2) **15** *A. egina* (4) **16** *A. omrora* (4) **17** *A. nohara* (4) **18** *A. oncaea* (3) **19** *A. zetes* (4) **20** *A. endoscota* (4) **21** *A. quirina* (3) **22** *A. igati* (3) **23** *A. hamata* (2). Scale bar = 1 mm.

A total of 273 butterflies species in two families – Papilionidae (72 species, 13 genera) and Nymphalidae (201 species, 9 genera) – were recorded as having a sphragis, protosphragis, vestigial sphragis, or hemi-sphragis (Suppl. material 1) (Figures 1–33). These numbers represent 13% of Papilionidae, 3% of Nymphalidae, and 1% of all butterflies (Heppner 1991, Häuser et al. 2005). The 22 sphragis-bearing genera were distributed in 8 tribes within the two families. A well-formed sphragis occurrs in almost all species of *Parnassius*, where it is probably plesiomorphic, and was widespread in *Acraea* (ca 64% of species in the genus). On the other hand, in *Heteronympha* (Figure 13), it occurs in just one out of eight species. The presence of a sphragis in the Ithomiini *Pteronymia* remained undetected until recently (De-Silva et al. *in press*), probably due its small size when compared to other groups, which is consistent with the slim build and small size of butterflies in this genus.

A protosphragis was present in 11 species, vestigial sphragides occurred in 14 species, while a hemi-sphragis is found only in the two known species of the Troidini genus *Trogonoptera* (Suppl. material 1). A girdle was present, at least facultatively, in 23 species and was widespread in some *Parnassius* species-groups, such as in *delphius* (Figure 3) and *acco* groups.

For the species where a sphragis occurs (Suppl. material 1), Category 3 (99 species) and Category 4 (98 species) were most common, together being found in 197 species (72%), whereas Category 1 was least represented, with 25 species (9%) (Table 2). We could not define a category for 16 sphragis-bearing species due to lack of data, which in most cases was due to the small number of specimens available, preventing a confident determination on the complexity of the sphragis. Although most species fit well within our categories, intermediates occurred. Examples include *Allancastria* spp. (Papilionidae: Parnassiinae), in which the sphragis might best be characterized as falling between Categories 2 and 3, as well as *Amauris niavius* (Linnaeus, 1758) (Nymphalidae: Danainae) (Figure 12) and *Losaria palu* (Martin, 1912) (Papilionidae: Papilioninae), which could be considered to fall between Categories 1 and 2. We listed these species in Suppl. material 1 based on which category their sphragis best fits. The fact that complex sphragides are more common across butterfly groups might indicate that male adaptations to produce them are subject to strong selection.

**Table 2.** Number of species per sphragis category.

	Category 1	Category 2	Category 3	Category 4	Uncertain
Number of species	25	35	99	98	16
Percentage of species	9%	13%	36%	36%	6%

### Variation in the morphology and color of the sphragis

Four categories of sphragis (including two subcategories) were recognized in terms of structural complexity (Suppl. material 1 and Table 2). As an external, large, and morphologically complex sphragis, there is great variation in size, color and shape (Figures 1–33). For example, the sphragis in species of the *P. mnemosyne* species-group is an enormous, hollow, thin-walled tubular structure, nearly as large as the female abdomen (Figure 5). Our characterizations show that while sphragides are frequently solid, hollowness is relatively common (27%) (Table 3). This could be a strategy to maximize the bulk of the sphragis without increasing mass (Orr 1988), thus optimizing its effectiveness while reducing the material cost to the male in its production, as well as reducing the load carried by the female. Such forms are associated with major adaptations in the male genitalia (Orr 1995). The sphragis of most *Parnassius* species is generally hollow and those of the *P. mnemosyne* group (Figure 5) include the largest sphragides so far measured (in terms of total mass). Based on available data, the sphragis of *P. glacialis* Butler, 1866 represents approximately 20.5% of male dry weight (Orr 1988) (Table 1). Those of *P. imperator* Oberthür, 1883 (Figure 4), *P. charltonius* Gray, (1853) (Figure 2) and *P. acco* Gray, (1853) may be even heavier in relative terms (Orr 1988).

**Table 3.** Number of species displaying different sphragis parameters.

Condition	No. of species
Scales	
Yes	145
No	113
Projections and/or girdle	
Yes	60
No	199
Structure	
(Mostly) Hollow	73
(Mostly) Solid	176

*Acraea* species exhibit great variation in the form and development of the sphragis. In some, such as *A. natalica* Boisduval, 1847, there is no sphragis or internal mating plug and females mate numerous times with males, which produce small spermatophores (Orr 1988). The form of the female genitalia and the small size of the spermatophore suggests that this species evolved from sphragis-bearing ancestors. Similarly, the sphragis has probably been lost in *A. encedon* (Linnaeus, 1758) and its relatives (Pierre 1985b), but in these species there is an internal plug. The sphragis has therefore apparently been lost independently at least twice in African *Acraea*; in other species the sphragis may be vestigial. In the Acraea subgenus Actinote, females typically bear a small to medium sized sphragis covered in male scales (Figure 32) (Pierre 1985a). These sphragides tend to be relatively small compared to male mass (see *A. serena* (Fabricius, 1775) in Table 1). Other *Acraea* species bear a medium to large, box-like hollow sphragis (Figure 20), produced in a similar manner to those of *Parnassius* (Orr 1988). These seem to represent a larger investment by the male (see *A. anemosa* Hewitson, 1865 in Table 1) and this is probably true of most hollow sphragides in *Acraea*. Small sphragides incorporating dense, long scales have also been described in *Allancastria* species (Matsumoto et al. *in prep*.) (Papilionidae: Parnassiinae). A medium-sized, scale-covered structure occurs in the *H. penelope*, which produces a largely hollow sphragis (in Figure 13, the sphragis is probably from the male’s second mating) (Orr 2002). Numerous scales are incorporated into the large twisted sphragis of *Luedorfia puziloi* (Erschoff, 1872) (Figure 9) and also in the medium-sized flat, shield-like sphragis of *Luehdorfia japonica* Leech, 1889 (Matsumoto 1987). Some medium sized sphragides are probably extremely effective in preventing remating. For example, the sphragis of *C. cressida* is solid with lateral projections, a dorsal keel, and an anterior horn (Orr 1999b), molded within a system of membranes in the male genitalia and enclosed by valves. It represents about 6.7% of male mass, but is very effective in preventing remating (Orr 1988). The sphragis of some species, notably *Euryades corethrus* (Boisduval, 1836) and *E. duponchelii* (Lucas, 1836) have long projections (Figure 11). This type of sphragis, formed in deep sheaths within the male’s body (Figure 34) (Miller 1987), potentially make it difficult for subsequent males to grasp the body of mated females to remove the sphragis. In *E. corethrus*, the total mass of the structure is a moderate 8.6% of male body mass (Orr 1988), suggesting efficient use of material. On the other hand, solid small-medium size sphragides lacking scales occur in many *Acraea* species. In these cases the sphragis is formed in a sclerotized mold associated with the male genitalia (van Son 1963, Orr 1988). In *A. andromacha* the sphragis represents 3.1% of male body mass (Table 1), but is not completely effective in preventing remating, with sphragis removal and limited polyandry having been reported (Epstein 1987, Orr 1988).

**Figures 24–33.**
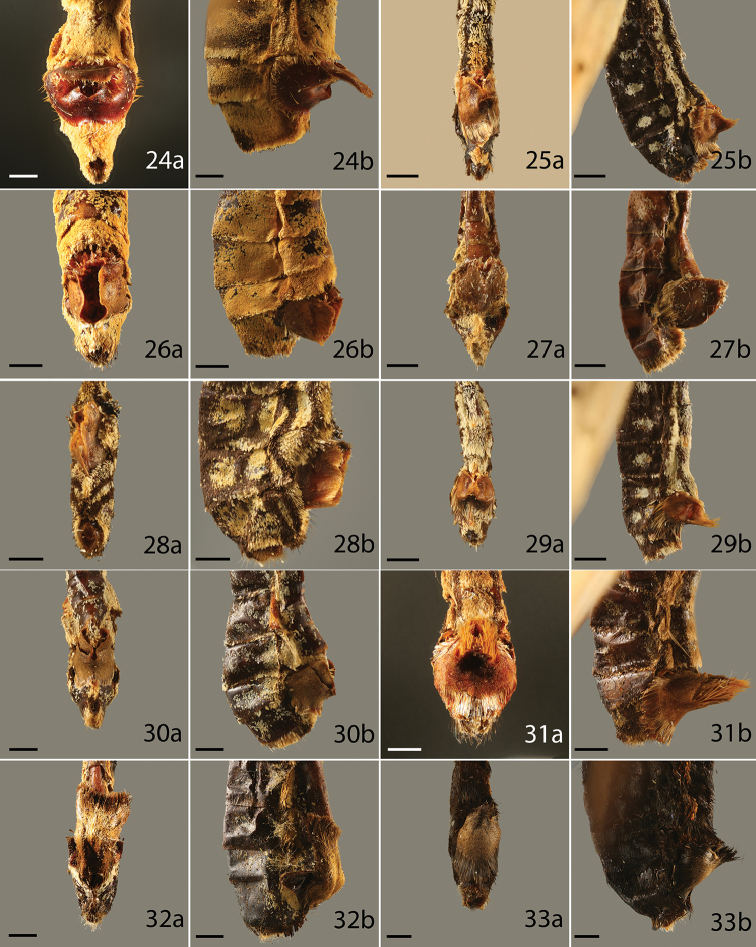
Sphragis of butterfly species, **a** ventral **b** lateral, category of the sphragis in parenthesis. **24** *Acraea umbra* (4) **25** *A. quirinalis* (3) **26** *A. pharsalus* (2) **27** *A. serena* (3) **28** *A. althoffi* (3) **29** *A. orestia* (3) **30** *A. pentapolis* (3) **31** *A. issoria* (3) **32** *A. rhodope* (3) **33** *A. ozomene* (3). Scale bar = 1 mm.

A girdle occurs in *E. corethrus* (but not in the related *E. duponchelii*) (Bryk 1918) and in several *Parnassius* species such as *P. autocrator* Avinov, 1913 (Figure 1), *P. delphius* (Eversmann, 1843) (Figure 3), and *P. imperator* (Figure 4). It occurs in *P. cephalus* Grum-Grshimailo, 1891, but not in its sister species *P. szechenyii* Frivaldszky, 1886, possibly because the final segment of the abdomen to which the sphragis is attached is unusually deep, making it impossible for the male to encircle the abdomen with his valves (Orr 1988). Recent phylogenies of *Parnassius* (Michel et al. 2008, Omoto et al. 2009) suggest that the girdle has evolved independently at least three times within the genus. The girdle present in a few *Acraea* appears to be a facultative condition, possibly associated with a male’s first mating. It loosely encircles the abdomen, but evidently does not grip it tightly above as in girdled papilionid species. The variation in color of the sphragis might reflect differences in the composition of the sphragidal material; this subject deserves further investigation. In the *P. apollo* group (*phoebus*, Figure 6), sister to the remainder of the genus, it is brown and slightly translucent whereas in other species it is whitish (often discolored) and opaque.

Almost all butterfly species in our list display structures that meet our definition of the sphragis, however, we recognized a few unusual intermediate forms. The large, semi-exposed plug of the troidine papilionid *Trogonotera* Rippon, 1890 has internal structure (ordered lacunae) and external, well defined striae, hence we classify it as a ‘hemi-sphragis’. The male has a specialized pouch where the plug material is formed into a broad ribbon, which coalesces into a solid body with lacunae (Orr 1988, 1995). The small formation of the troidine papilionid *Parides proneus* (Hübner, 1831), recognized by Bryk (1918) as a true sphragis, is regular in shape but is fixed to a bar over the female ostium (Table 1) (Bryk 1918). This structure is comparable in size with the sphragis of many *Acraea* species. The hemi-sphragis of *Trogonoptera* and the small sphragis of *P. proneus* both have an intermediate plug/spermatophore ratio, with approximately 40% of male secretions being allocated to the sphragis, but the remainder being allocated to the spermatophore and spermatophylax, a granular secretion filling the appendix bursae (Orr 1988, 1995). This intermediate condition is unusual. Species that produce an internal plug typically allocate less than 30% of male accessory gland secretion to the plug, versus 70% to the spermatophore (n=50 species; x–=14.8; s=9) (Orr 1988, 1995). However, sphragis-bearing species allocate between 72% and 99% of accessory secretions to the sphragis, versus less than 30 percent to the spermatophore (n=34 species; x–= 84.1; s=16), resulting in a bimodal frequency distribution of material allocated to sperm guarding (Orr 1988, 1995).

Although the sphragis appears to be an effective structure to prevent remating, there are examples where the female might be able to mate again (Pierre 1985a, Epstein 1987, Matsumoto 1987, Orr 1988, Sourakov and Emmel 1997). For example, a second mating might occur soon after the first mating, while the sphragis of the first male is still relatively soft and can be moved or removed by the second male, or if the first sphragis was unusually frail. Sourakov and Emmel (1997) reported a male of *A. epaea* (Cramer, 1779) on top of a female while she mated with another male, presumably so he too could mate with her. This however, might not guarantee success to the second male; Orr (1988) figured a female of *A. serena* bearing three sphragides congealed into a single mass that incorporated the spermatophores of the second and third males, and which could not possibly have fertilized the female due to the presence of the first sphragis blocking the copulatory opening. In general it is important to appreciate that the presence of a double sphragis, (termed a plethosphragis by Bryk (1924)), does not imply that the second male was able to inseminate that female because as long as the original sphragis remains intact, the spermatophore of the second male remains outside the body of the female. Allowing time for the sphragis to harden could be the reason for protracted mating in *C. cressida*, in which the pair remains coupled for 17 hours or more (Orr 1988).

**Figure 34.**
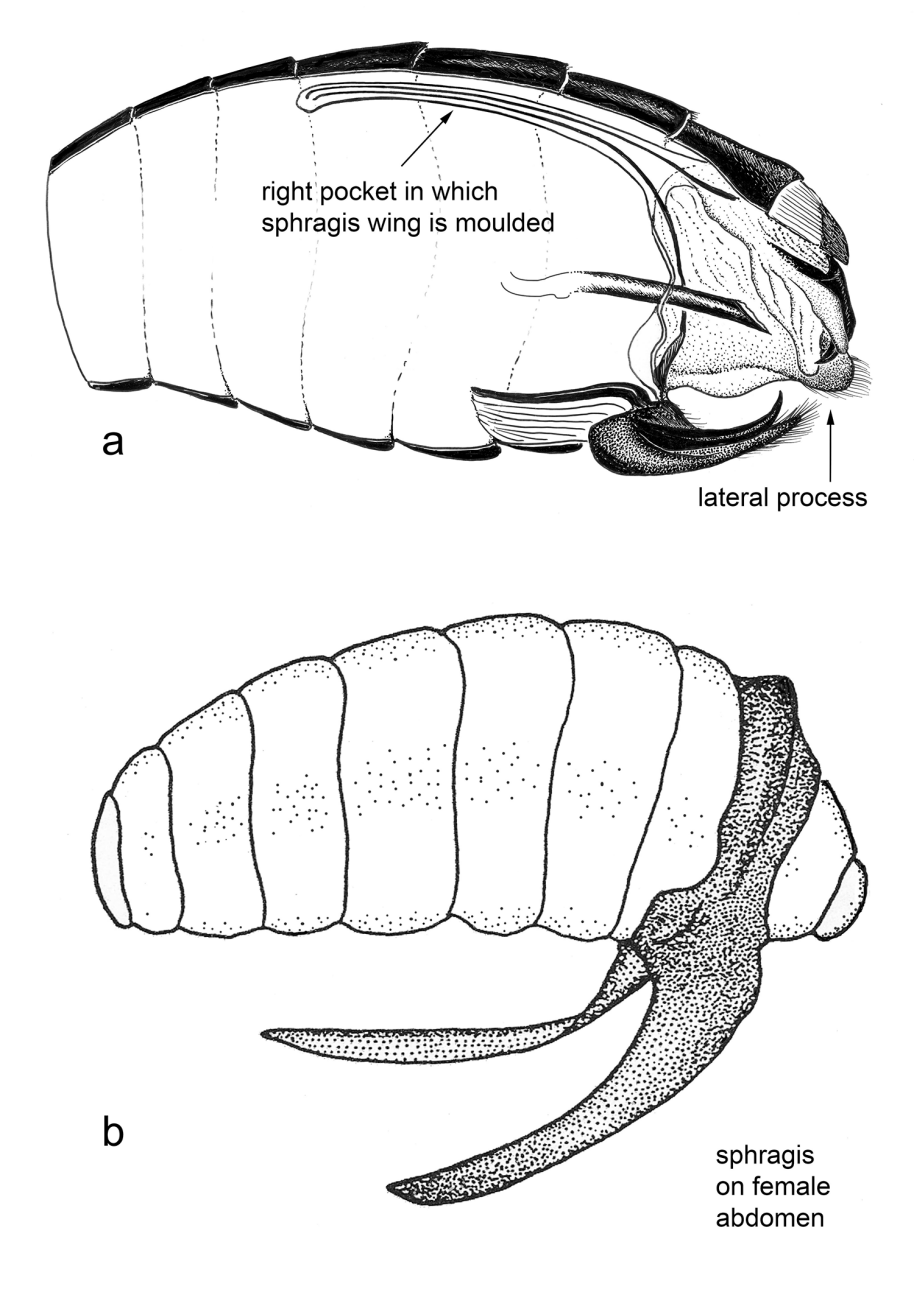
Example of male adaptations associated with sphragis production: **a** parasagittal section of *Euryades corethrus* male showing deep pockets where sphragis wings are molded and other features associated with sphragis production **b** The finished sphragis *in situ* on the female abdomen.

There are cases known in *Parnassius* species where hardened sphragides have been lost by the female or were removed by subsequent males (Matsumoto and Suzuki 1995, Vlasanek and Konvicka 2009), which would potentially allow additional matings. Moreover, Orr (1988) experimentally induced males of *C. cressida* to mate with caged mated females, and they eventually dissolved the attached sphragis of the first male. However, he noted that the energy and time spent removing the sphragis (around 30 hours) makes this an unlikely occurrence in nature if the sphragis is well formed and would potentially result in the death of one or both butterflies. This process is probably only used as an aid to remove frail sphragides deposited by depleted males (Orr 1999b).

The satyrine nymphalid *H. penelope* is the only species of its genus, and subfamily, bearing a true sphragis (Figure 13) (Orr and Kitching 2010). It does not correspond with all morphological and behavioral adaptations found in other sphragis-bearing butterflies. The external female genitalia are virtually unmodified from the condition found in other species of the genus, whereas all other sphragis-bearing species exhibit profound modifications in this structure, especially exhibiting externalization of the ostium (Orr 2002). However, the highly modified male genitalia are efficient at plug removal. Males bear androconial patches on the wings and exhibit courtship behavior (characteristics often found in polygynous species (Pliske 1975)), although they may facultatively practice aerial capture and forced copulation (Orr 2002).

### Towards an understanding of sphragis evolution

**Figure 35.**
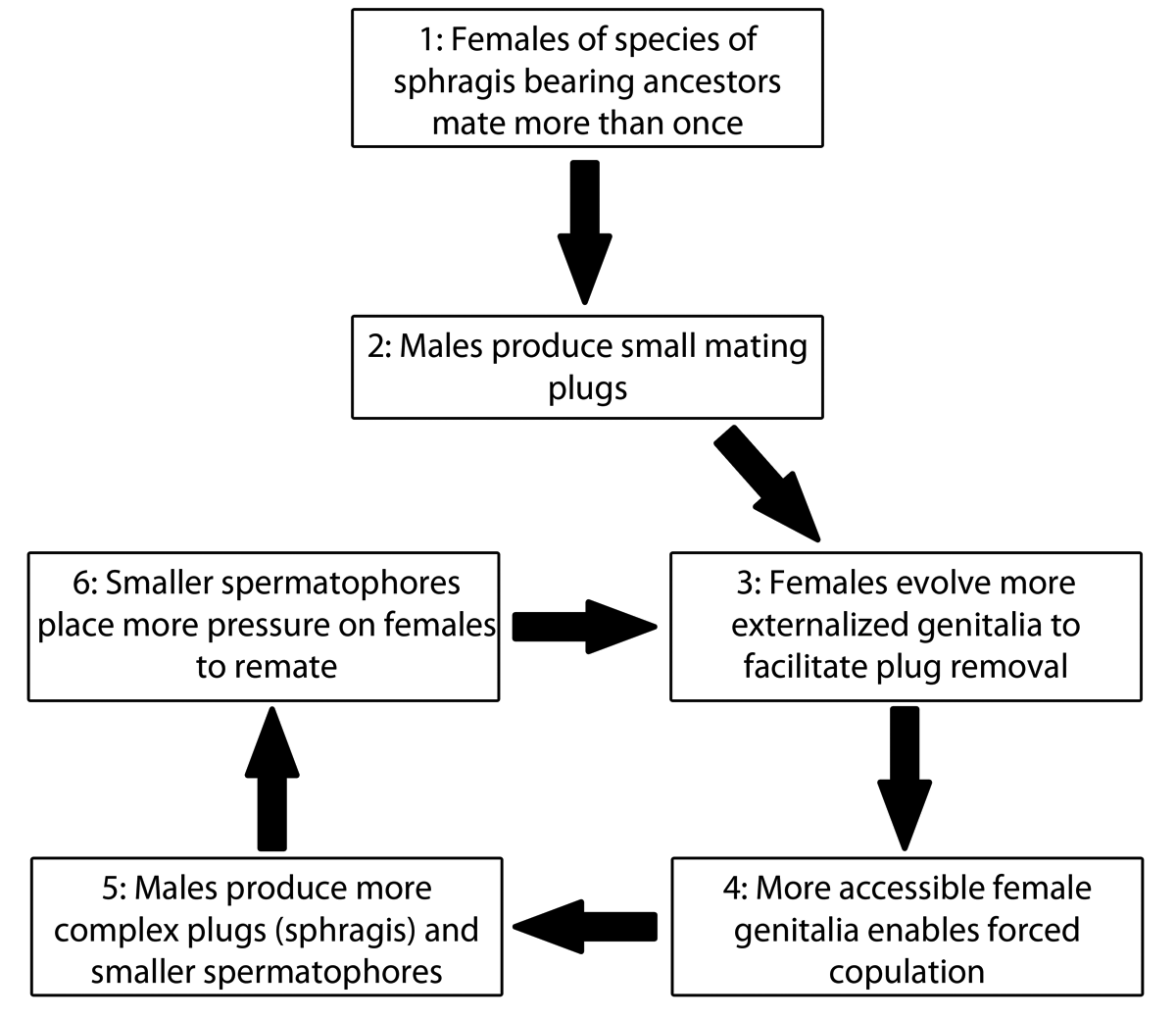
Schematic of the possible process of evolution of the sphragis in butterflies. This assumes a selective landscape where females benefit materially from polyandry and males are continually improving plug-removing ability.

The evolution of the sphragis was studied in detail by Orr (1988, 1995, 1999b, 2002) and Matsumoto (1987, Matsumoto and Suzuki 1995). Orr’s studies utilized a comparative analysis of genital morphology, reproductive physiology, and behavior in a wide range of sphragis-bearing species. Non-sphragis bearing butterflies in Papilionidae, Pieridae and Nymphalidae were examined for outgroup comparison. Based on an analysis of convergent traits in unrelated lineages, Orr’s hypothesis for sphragis evolution is as follows (Figure 35): (1) females of the ancestral, non-sphragis bearing butterfly species benefited from mating with more than one male, most likely due to enhanced material benefits, chiefly protein, gained from the spermatophore; (2) males produced small mating plugs to prevent female remating; (3) females evolved more externalized genitalia (Orr 1988, 1995) (Type 1 of Orr (1995)) that made small mating plugs attached to the spermatophore ineffective and easily removed by males, which simultaneously evolved better mechanisms for physically removing plugs; (4) externalizing the female genitalia made their genitalia more accessible, enabling males to copulate by force, a behavior almost universal in sphragis-bearing species, that increased the chance of female remating; (5) males also responded by producing larger and more complex plugs (sphragides) at the expense of spermatophore size, thereby (6) increasing the pressure on females to mate more than once as the nutritional contribution by males per mating diminished. This led to an escalated arms race where female genitalia became more and more externalized, making it more difficult to affix a sphragis, and more heavily armored to protect them from injury during mating attempts that involved the violent removal of a sphragis. Male genitalia became more specialized to produce sphragides that could not be removed, as they reduced their nutritional contribution via the spermatophore, at the same time increasing plug-removing abilities. The female bursa copulatrix became smaller accordingly, leading to the extreme case where it has completely atrophied in *C. cressida*. This is the ‘male wins’ scenario, which leads to obligate sphragis formation. Alternatively, males of some species were unable to produce an effective sphragis to counter female anti plugging strategies and so ceased to plug at all and mated as often as possible, a ‘female wins’ scenario. Orr (1988) suggested this is possibly occurring in certain danaines and also the hyper polyandrous *Acraea natalica*. Part of the reason for this runaway process and the instability of intermediate conditions may lie in the asymmetry of male and female adaptations (Orr 1988).

**Figure 36.**
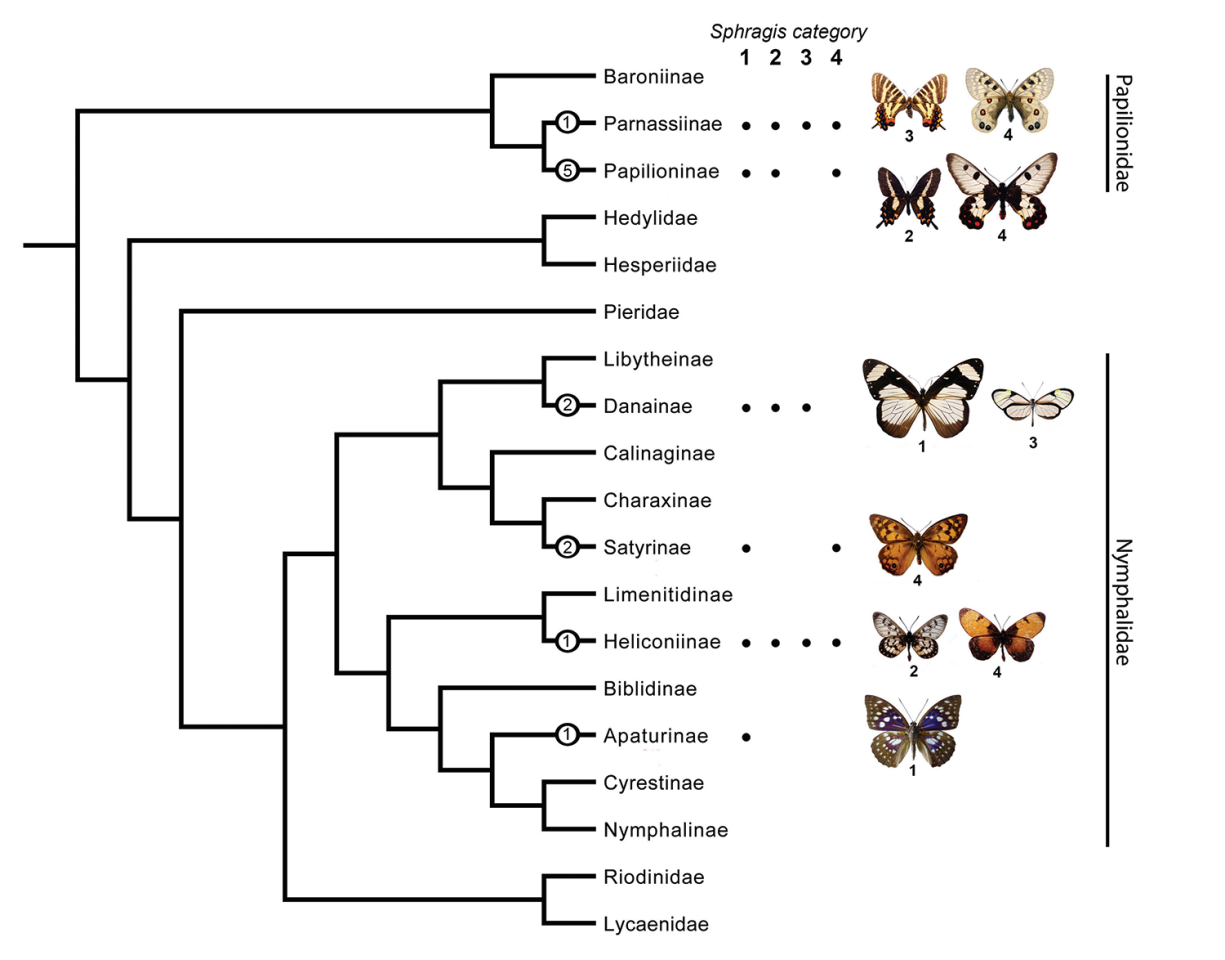
The occurrence of the sphragis in butterfly subfamilies. Dark circles indicate that some species in the clade bear sphragides or a version of it. Numbers inside the dark circles indicate estimation of minimum number of sphragis evolution events. Numbers under butterfly images indicate sphragis category of that species. Tree adapted from the phylogeny of Heikkila et al. (2011).

**Figure 37.**
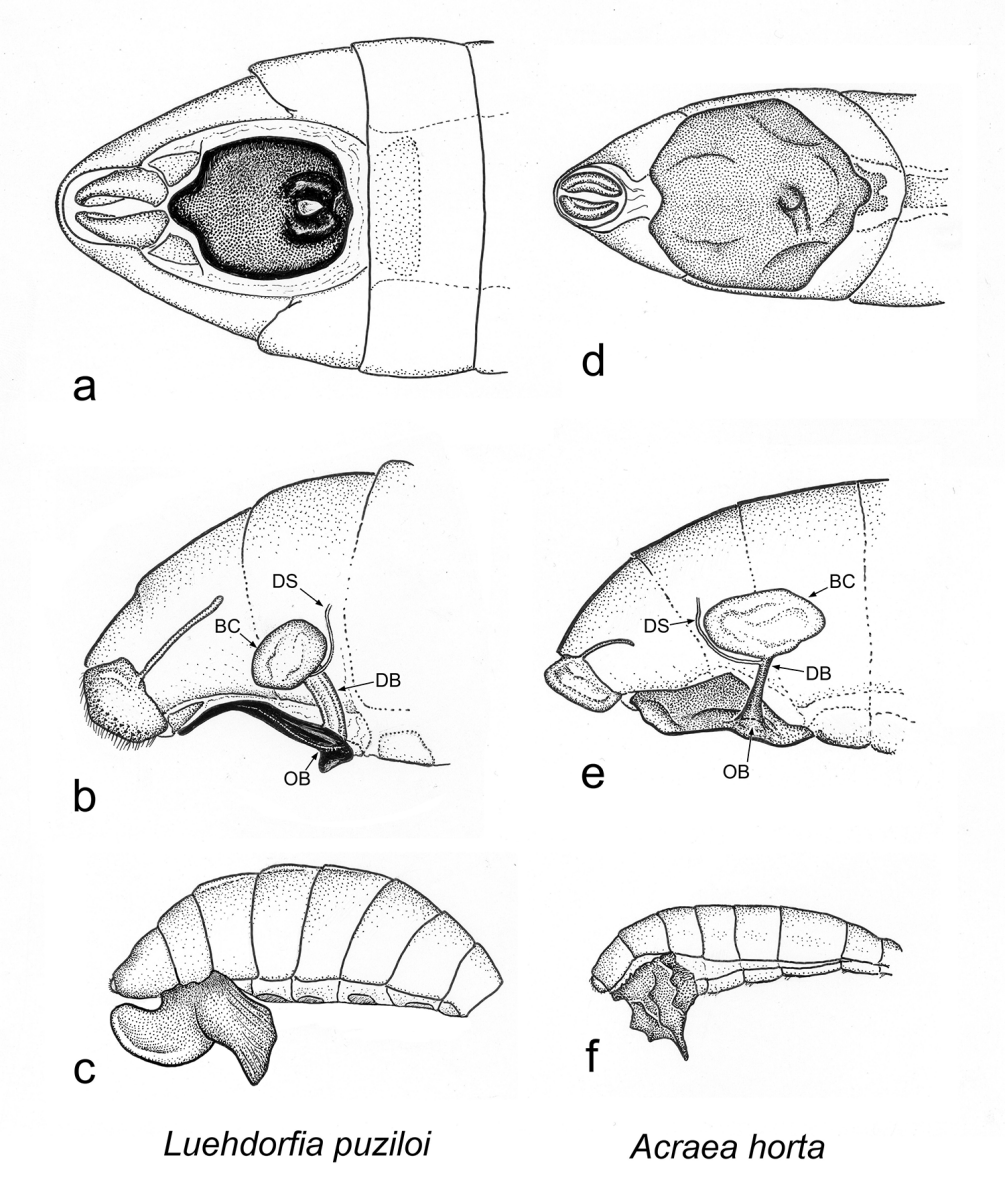
Ventral and parasagittal views of the female genitalia and lateral abdomen with sphragis for *Luehdorfia puziloi* (**a, b, c** respectively), and *Acraea horta* (**d, e, f** respectively), showing convergence in externalization of female genitalia, reduction in the size of the bursa copulatrix, and how the genitalia is covered by the sphragis. BC, bursa copulatrix, DB, ductus bursae; DS, ductus seminalis; OB, ostium bursa.

Furthermore, Orr (1995) suggested that the sphragis evolved independently at least two times in the Papilionidae and two times in Nymphalidae, but recent phylogenetic studies (Braby et al. 2005, Condamine et al. 2012, Simonsen et al. 2012) indicate that eight times across the two butterfly families may be a better minimum estimate for independent sphragis evolution at level 2 or higher (Figure 36). This is supported by qualitative differences in the sphragis between papilionids and some *Acraea* species, as well as highly specialized external female genitalia evolving non-homologous elements (Orr 1988, 1995) in the two families (Figure 37).

When we include Category 1 in our analysis, the sphragis appears to have arisen in at least six subfamilies in butterflies (Figure 36) and deeper analysis within subfamilies would potentially double this number. Within each of these groups, some species appear to have lost the sphragis completely. For instance, the *Parnassius simo* group is the only species group in *Parnassius* that lacks a sphragis. All species of *Zerynthia* (Papilionidae: Parnassiinae) appear to be in the process of losing the sphragis with vestiges found in some individuals (Matsumoto et al. in prep). Among the 287 described (Pierre and Bernaud 2014b) *Acraea* species, at least 183 have a sphragis or a sphragis-like structure although it is unclear whether the sphragis is plesiomorphic for the genus (Pierre 1985a). Within *Acraea*, the *A. encedon* species-group (subgenus Actinote) and *A. natalica* (subgenus Acraea), are two of several candidates that have apparently lost the sphragis. Phylogenetic analyses of sphragis-bearing taxa, and their close relatives lacking a sphragis, may reveal whether particular cases are plesiomorphic or derived.

The two most complex forms of the sphragis (Categories 3 and 4) are found in five out of six subfamilies where the sphragis is found (Figure 36). It could be an indication of strong convergent evolution and positive selection force for the development of complex sphragis structure along butterflies.

In a final twist of the hypothesized evolutionary process, it is possible that in some circumstances the sphragis has become secondarily advantageous to females. Females of *L. japonica* usually mate only once, early in their life (Matsumoto 1987); the same occurs in *E. corethrus* (Nicolás Mega pers. com.) and *C. cressida* (Orr and Rutowski 1991, Orr 1999b). *Cressida* females derive no amino acid nutrients from the miniscule spermatophore (Orr 1988). In these cases, we can wonder if there is any aspect of female choice in these species and how the sphragis has helped this condition. Could females be “losing” in the arms race resulting from the intersexual conflict caused by the sphragis? Can it affect the genetic diversity of the species, considering that females cannot choose the most fit male? Bearing in mind that virgin females do escape copulation attempts (Orr 1999b), is there any feature of forced copulation that allows females to evaluate male sperm quality? These questions should be addressed to fully understand the effect of the sphragis on butterfly sexual dynamics. For some groups, the presence of the sphragis might be advantageous to females, especially if they receive enough sperm in one copulation and there is no transfer of nutritional substances from the male (Orr 1988). In the case where males visually detect mated females, the sphragis might prevent a male’s mating attempts, which are known to interfere with oviposition and can also cause physical damage to the female (Thornhill and Alcock 1983, Orr 1999b).

It is known that in Papilionidae extremely complex female genitalia occur widely, as well as the numerous externalized forms normally associated with bearing a sphragis (Miller 1987, Orr 1988). Complex female genitalia also occur to a lesser extent in Nymphalidae (Orr 1988), but not in the Hedylidae, Hesperiidae, Riodinidae, or Lycaenidae. Pieridae, as well as the papilionid genus *Battus* Scopoli, 1777, have pilose lobes flanking the ostium (Miller 1987, Orr 1988), which, at least in pierids, appear to be associated with the reception of antiaphrodisiacs from the male (Andersson et al. 2000, 2003, Schulz et al. 2008, Malouines 2016). These may largely obviate the need for mating plugs. A similar phenomenon occurs in *Heliconius* (Gilbert 1976). Data on the incidence of internal mating plugs and mating frequency in butterflies and moths are still too fragmentary for detailed analysis, but available evidence suggests that mating plugs are most common in groups with complex female genitalia. We expect that sphragis formation could only evolve in groups preadapted by having the capacity to produce a large internal mating plug. In general, the positive feedback process (Figure 35) leading to sphragis formation requires female genitalia to become externalized. However, complex genitalia present other possibilities. For example, it has been suggested that some female genital structures may help grip the mating plug (Orr 1988), allowing the male to make a large investment in a nutritious spermatophore, with confidence that a smaller plug would suffice to protect his paternity. If such processes are occurring, it is perhaps not surprising that the sphragis occurs relatively rarely, given that sexual conflict may be resolved in other ways. This is especially likely considering that the sphragis may come at a cost to the species in terms of reduced female fecundity and the disadvantages accruing from the encumbrance of the sphragis itself and attacks from rapacious males. It is possible however that by studying this phenomenon, we may better understand the intersexual dynamics of mating systems in butterflies generally.

Many questions still remain unanswered, especially regarding the processes involved in the evolution of the sphragis. Additionally, more studies are necessary to investigate how the presence of the sphragis may be related to factors such as ecology, especially habitat type and hostplant dispersion, reproductive behavior, and sperm dynamics, however, these topics go beyond the scope of the present study.

## Conclusions

This is the first near complete survey of variation and morphological complexity of the sphragis in butterflies to date. This study provides the most comprehensive review on the sphragis, besides suggesting a method of categorization of the structure.

The main outcomes are as follows:

1. A true sphragis was found in 232 species of butterflies from the families Papilionidae and Nymphalidae.

2. The sphragis and related structures can be categorized into roughly four structural types: low complexity (amorphous and facultative), moderate complexity, medium complexity, and high complexity.

3. The sphragis and related structures is found in the Papilionidae subfamilies Parnassiinae and Papilioninae, as well as in the Nymphalidae subfamilies Danainae, Heliconiinae, Apaturinae, and Satyrinae.

4. Based on previously published evidence, the sphragis functions primarily to prevent remating by a secondary male.

## Supplementary materials

Supplementary material 1 Table S1. Sphragis-bearing butterfly species and morphological characteristics of their sphragides Data type: Microsoft Excel document Explanation note: G: girdle, VS: vestigial sphragis, PS: protosphragis, HS: hemi-sphragis, *: species is figured in the plates. This dataset is made available under the Open Database License (http://opendatacommons.org/licenses/odbl/1.0/). The Open Database License (ODbL) is a license agreement intended to allow users to freely share, modify, and use this Dataset while maintaining this same freedom for others, provided that the original source and author(s) are credited.Ana Paula S. Carvalho, Albert G. Orr, Akito Y. Kawahara

## References

[B1] Ackery PR (1975) A guide to the genera and species of Parnassiinae (Lepidoptera: Papilionidae). Bulletin of the British Museum 31: 71–105. https://doi.org/10.5962/bhl.part.29484

[B2] Arnqvist G Nilsson T (2000) The evolution of polyandry: multiple mating and female fitness in insects. Animal Behaviour 60: 145–164. https://doi.org/10.1006/anbe.2000.1446 10973716 10.1006/anbe.2000.1446

[B3] Beccaloni G Scoble M Kitching I Simonsen T Robinson G Pitkin B Hine A Lyal C (2003) The Global Lepidoptera Names Index (LepIndex). World Wide Web electronic publication. Available from: http://www.nhm.ac.uk/our-science/data/lepindex/ [October 1, 2016]

[B4] Bingham CT (1907) The Fauna of British India, including Ceylon and Burma, Butterflies Vol. 2. Taylor and Francis, London, 480 pp https://doi.org/10.1038/115115c0

[B5] Blanckenhorn WU Hosken DJ Martin OY Reim C Teuschl Y Ward PI (2002) The costs of copulating in the dung fly *Sepsis cynipsea*. Behavioral Ecology 13: 353–358. https://doi.org/10.1093/beheco/13.3.359

[B6] Boggs CL Gilbert LE (1979) Male Contribution to Egg Production in Butterflies: Evidence for Transfer of Nutrients at Mating. Science 206: 83–84. https://doi.org/10.1126/science.206.4414.83 17812454 10.1126/science.206.4414.83

[B7] Boggs CL Watt WB (1981) Population structure of pierid butterflies IV. Genetic and physiological investment in offspring by male *Colias*. Oecologia 50: 320–324. https://doi.org/10.1007/BF00344970 28309048 10.1007/BF00344970

[B8] Bollino M Racheli T (2012) Butterflies of the World, Supplement 20. Parnassiinae (Partim) Parnassiini (Partim); Luehdorfiini; Zerynthiini (Lepidoptera: Papilionidae). Goecke & Evers, Keltern, 64 pp.

[B9] Braby MF Trueman JWH Eastwood R (2005) When and where did troidine butterflies (Lepidoptera: Papilionidae) evolve? Phylogenetic and biogeographic evidence suggests an origin in remnant Gondwana in the Late Cretaceous. Invertebrate Systematics 19: 113–143. https://doi.org/10.1071/IS04020

[B10] Bryk F (1918) Grundzüge der Sphragidologie. Arkiv för Zoologi 11: 1–38.

[B11] Bryk F (1919) Bibliotheca sphragidologica. Archiv für Naturgeschichte 85: 102–183.

[B12] Bryk F (1924) Über die Disphragophorie der Schmetterlingsweibchen. Societas Entomologica 12: 45–47.

[B13] Bryk F (1930) Monogame Einrichtungen bei Schmetterlingsweibchen. Archiv für Frauenkunde und Konstitutionsforschung 16: 308–313.

[B14] Bryk F (1935a) Parnassiidae Pars II. [Parnassiidae Part II]. In: Schulze F, Kükenthal W, Heider K, Hesse R, Abstein C (Eds), Das Tierreich 65. W. de. Gruyter, Berlin, 790 pp.

[B15] Bryk F (1935b) Sericinus sphragidophor! Parnassiana 3: 75.

[B16] Bryk F (1950) Geographische und individuelle Variabilität der Sphragisbildung (Lepidoptera: Parnassiidae). Entomologisk tidskrift 71: 230–234.

[B17] Callahan PS Cascio T (1963) Histology of the Reproductive Tracts and Transmission of Sperm in the Corn Earworni, *Heliothis zea*. Annals of the Entomological Society of America 56: 535–556. https://doi.org/10.1093/aesa/56.4.535

[B18] Chapman RF (1969) The Insects: structure and function. Cambridge University Press, Cambridge, 959 pp.

[B19] Chapman T Patridge L (1996) Sexual conflict as fuel for evolution. Nature 381: 189–190. https://doi.org/10.1038/381189a0 8622753 10.1038/381189a0

[B20] Churkin S (2006) A new species of *Parnassius* Latreille, 1804 from Kyrgyzstan (Lepidoptera, Papilionidae). Helios 7: 142–158.

[B21] Common IFB Waterhouse DF (1981) Butterflies of Australia. Angus and Robertson, Melbourne, xiv, 682 49 of plates pp.

[B22] Condamine FL Silva-Brandão KL Kergoat GJ Sperling FAH (2012) Biogeographic and diversification patterns of Neotropical Troidini butterflies (Papilionidae) support a museum model of diversity dynamics for Amazonia. BMC Evolutionary Biology 12. https://doi.org/10.1186/1471-2148-12-82 10.1186/1471-2148-12-82 PMC3464124 22690927

[B23] D’Abrera B (1984) Butterflies of the Neotropical Region: Pt. 2: Danaidae, Ithomiidae, Heliconidae & Morphidae. Hill House Publishers, Malvern, 384 pp.

[B24] D’Abrera B (1990) Butterflies of the Holarctic Region: Papilionidae, Pieridae, Danaidae & Satyridae (Partim) Pt. 1. Hill House Publishers, Melbourne, 185 pp.

[B25] D’Abrera B (1995) Butterflies of the Neotropical Region: Brassolidae, Acraeidae, Nymphalidae (Partim) Pt. 3. Hill House Publishers, Melbourne, 147 pp.

[B26] D’Abrera B (1997) Butterflies of the Afrotropical Region: Papilionidae, Pieridae, Acraeidae, Satyridae Pt. 1. Hill House Publishers, Melbourne, 613 pp.

[B27] Drummond BA (1984) Multiple mating and sperm competition in the Lepidoptera. In: Smith RL (Ed.) Sperm competition and the evolution of animal mating systems. Academic Press, London, 291–370. https://doi.org/10.1016/B978-0-12-652570-0.50016-6

[B28] Eaton JL (1988) Lepidopteran Anatomy. Wiley-Interscience, Hoboken, 257 pp.

[B29] Ehrlich AH Ehrlich PR (1978) Reproductive Strategies in the Butterflies: I. Mating Frequency, Plugging, and Egg Number. Journal of the Kansas Entomological Society 51: 666–697.

[B30] Ehrlich PR (1961) Comparative morphology of the male reproductive system of the butterflies (Lepidoptera: Papilionoidea). 1. Some nearctic species. Microentomology 24: 135–166.

[B31] Eltringham H (1912) A Monograph of the African species of the Genus *Acraea*, Fab., with a supplement on those of the Oriental Region. Transactions of the Royal Entomological Society of London 60: 1–369. https://doi.org/10.1111/j.1365-2311.1912.tb02511.x

[B32] Eltringham H (1925) III. On the Source of the Sphragidal Fluid in *Parnassius apollo* (Lepidoptera). Transactions of the Royal Entomological Society of London 73: 11–15. https://doi.org/10.1111/j.1365-2311.1925.tb02859.x

[B33] Epstein ME (1987) Mating behavior of *Acraea andromacha* (Fabricius) (Nymphalidae) in New Caledonia. Journal of the Lep 41: 119–121.

[B34] Ferro DN Akre RD (1975) Reproductive Morphology and Mechanics of Mating of the Codling Moth, *Laspeyresia pomonella*. Annals of the Entomological Society of America 68: 417–424. https://doi.org/10.1093/aesa/68.3.417

[B35] Francini RB Freitas AVL Penz CM (2004) Two new species of *Actinote* (Lepidoptera, Nymphalidae) from Southeastern Brazil. Zootaxa 719: 1–10.

[B36] Gilbert LE (1976) Postmating Female Odor in *Heliconius* Butterflies: A Male-Contributed Antiaphrodisiac? Science 193: 419–420. https://doi.org/10.1126/science.935877 10.1126/science.935877 935877

[B37] Gillies MT (1956) A new character for the recognition of nulliparous females of *Anopheles gambiae*. Bulletin of the World Health Organization 15: 451–459. 13404432 PMC2538298

[B38] Guenée MA (1872) Notice sur Divers Lépidoptères du Misée de Genève. Memoires de la Société de physique et d’histoire naturelle de Genève 21: 369–424.

[B39] Gwynne DT (1984) Male Mating Effort, Confidence of Paternity, and Insect Sperm Competition. In: Smith RL (Ed.) Sperm competition and the evolution of animal mating systems. Academic Press, London, 177–149. https://doi.org/10.1016/B978-0-12-652570-0.50011-7

[B40] Harada M Wangdi K Wangdi S Yago M Aoki T Igarashi Y Yamaguchi S Watanabe Y Sherub Wangdi R Drukpa S Saito M Moriyama Y Uchiyama T (2012) Rediscovery of Ludlow’s Bhutan Glory, *Bhutanitis ludlowi* Gabriel (Lepidoptera: Papilionidae): morphology and biology. Butterflies 10: 4–15.

[B41] Haude G (1913) Betrachtungen über den Zweck der Legetasche bei den Parnassierweibchen. Societas entomologica 28: 93–94.

[B42] Haüser CL de Jong R Lamas G Robbins RK Smith C Vane-Wright RI (2005) Papilionidae – revised GloBIS/GART species checklist (2nd draft). Available from: http://www.insects-online.de/frames/papilio.htm [October 1, 2016]

[B43] Heppner JB (1991) Faunal Regions and the Diversity of Lepidoptera Tropical Lepidoptera 2, supplem: 1–85.

[B44] Hinton HE (1964) Sperm transfer in insects and the evolution of haemocoelic insemination. In: Highnam KC (ed.) Insect Reproduction. Symposium of the Royal Entomological Society of London, 95–107.

[B45] Hirt K Ruch J Schneider JM (2017) Strategic male mating behaviour in *Argiope lobata*. Animal Behaviour 124: 27–34. https://doi.org/10.1016/j.anbehav.2016.11.030

[B46] Hosken DJ Stockley P Tregenza T Wedell N (2009) Monogamy and the battle of the sexes. Annual Review of Entomology 54: 361–378. https://doi.org/10.1146/annurev.ento.54.110807.090608 10.1146/annurev.ento.54.110807.090608 18793102

[B47] Houlbert C (1916) Contribution à l’étude das armatures génitales de deus Espèced malgaches appartenant au Genre *Acraea* (Lép. Nymphalidae). Lépidoptérologie Comparée: 135–172. Available from: http://www.biodiversitylibrary.org/item/41276.

[B48] Klowden MJ (2013) Physiological Systems in Insects. Elsevier Science, Amsterdam, 697 pp.

[B49] Knowlton N Greenwell SR (1984) Male Sperm Competition Avoidance Mechanisms: The Influence of Female Interests. In: Smith RL (Ed.) Sperm competition and the evolution of animal mating systems. Academic Press, London, 61–84. https://doi.org/10.1016/B978-0-12-652570-0.50009-9

[B50] Labine PA (1964) Population biology of the butterfly, *Euphydryas editha*. I. Barriers to Multiple Inseminations. Evolution 18: 335–336. https://doi.org/10.2307/2406408

[B51] Labine PA (1966) The Population Biology of the butterfly, *Euphydryas editha*. IV. Sperm precedence - A preliminary report. Evolution 20: 580–586. https://doi.org/10.1111/j.1558-5646.1966.tb03388.x 28562912 10.1111/j.1558-5646.1966.tb03388.x

[B52] Lamas G (2004) Checklist: Part 4A. Hesperioidea – Papilionoidea In: Heppner JB (Ed.) Atlas of Neotropical Lepidoptera Association for Tropical Lepidoptera, Gainesville.

[B53] Larsen TB (1991) The Butterflies of Kenya and Their Natural History. Oxford University Press, Oxford, 640 pp.

[B54] Larsen TB (2005) The Butterflies of West Africa. Apollo Books, New York, 865 pp.

[B55] Linnaeus C (1746) Fauna svecica, sistens animalia Sveciae regni. Laurentius Salvius, Stockholm, 411 pp.

[B56] Marshall GAK (1901) On the female pouch in *Acraea*. The Entomologist 34: 73–75.

[B57] Marshall GAK (1902) Five Years’ Observations and Experiments (1896–1901) on the Bionomics of South African Insects, chiefly directed to the Investigation of Mimicry and Warning Colours. Transactions of the Entomological Society of London 50: 287–584.

[B58] Matsumoto K (1987) Mating patterns of a sphragis-bearing butterfly, *Luehdorfia japonica* Leech (Lepidoptera: Papilionidae), with descriptions of mating behavior. Researches on Population Ecology 29: 97–110. https://doi.org/10.1007/BF02515428

[B59] Matsumoto K Suzuki N (1995) The nature of Mating Plugs and the probability of reinseminarion in Japanese Papilionidae. In: Scriber JM Tsubaki Y Lederhouse R (Eds) Swallowtail butterflies: their ecology and evolutionary biology. Scientific Publishers, Gainesville, 145–154.

[B60] Michel F Rebourg C Cosson E Descimon H (2008) Molecular phylogeny of Parnassiinae butterflies (Lepidoptera: Papilionidae) based on the sequences of four mitochondrial DNA segments. Annales de la Société entomologique de France (N.S. ) 44: 1–36. https://doi.org/10.1080/00379271.2008.10697541

[B61] Miller JS (1987) Phylogenetic Studies in the Papilioninae (Lepidoptera: Papilionidae). Bulletin of the American Museum of Natural History 186: 365–512.

[B62] Neild AFE (2008) The Butterflies of Venezuela, Part 2: Nymphalidae II (Acraeinae, Libytheinae, Nymphalinae, Ithomiinae, Morphinae). Meridian Publications, London, 275 pp.

[B63] Neild AFE Romero M (2008) Actinote Hübner [1819]. Species account. In: Neild AFE (Ed.) The Butterflies of Venezuela. Part 2: Nymphalidae II (Acraeinae, Libytheinae, Nymphalinae, Ithomiinae, Morphinae). A comprehensive guide to the identification of adult Nymphalidae, Papilionidae, and Pieridae. Meridian Publications, London, 25–46.

[B64] Omoto K Yonezawa T Shinkawa T (2009) Molecular systematics and evolution of the recently discovered “Parnassian” butterfly (*Parnassius davydovi* Churkin, 2006) and its allied species (Lepidoptera, Papilionidae). Gene 441: 80–88. https://doi.org/10.1016/j.gene.2008.10.030 19059318 10.1016/j.gene.2008.10.030

[B65] Orr AG (1988) Mate Conflict and the Evolution of the Sphragis in Butterflies. Griffith University.

[B66] Orr AG (1995) The evolution of the sphragis in the Papilionidae and other butterflies. In: Swallowtail butterflies, the ecology and evolutionary biology. Scientific Publisher Inc., Gainesville, 155–164.

[B67] Orr AG (1999a) Possible postcopulatory mate guarding in *Ornithoptera eupharion* (Gray) (Lepidoptera: Papilionidae). Australian Entomologist 26: 71–76.

[B68] Orr AG (1999b) The Big Greasy, *Cressida cressida* (Papilionidae). In: Kitching RL Scheermeyer E Jones RE Pierce NE (Eds) Biology of Australian Butterflies. Monographs on Australian Lepidoptera 6. CSIRO Publishing, Melbourne, 115–134.

[B69] Orr AG (2002) The sphragis of *Heteronympha penelope* Waterhouse (Lepidoptera: Satyridae): its structure, formation and role in sperm guarding. Journal of Natural History 36: 185–196. https://doi.org/10.1080/00222930010022926

[B70] Orr AG Kitching RL (2010) The Butterflies of Australia. Allen & Unwin, Crows Nest, 328 pp.

[B71] Orr AG Rutowski RL (1991) The function of the sphragis in *Cressida cressida* (Fab.) (Lepidoptera: Papilionidae): a visual deterrent to copulation attempts. Journal of Natural History 25: 703–710. https://doi.org/10.1080/00222939100770461

[B72] Paluch M Casagrande MM Mielke OHH (2003) Tampão genital de *Actinote* Hübner, como caráter taxonômico (Lepidoptera, Nymphalidae, Acraeinae). Revista Brasileira de Entomologia 47: 573–580. https://doi.org/10.1590/S0085-56262003000400007

[B73] Paluch M Casagrande MM Mielke OHH (2006) Três espécies e duas subespécies novas de *Actinote* Hübner (Nymphalidae, Heliconiinae, Acraeini). Revista Brasileira de Zoologia 23: 764–778. https://doi.org/10.1590/S0101-81752006000300022

[B74] Parker GA (1970) Sperm Competition and its evolutionary consequences in the Insects. Biological Reviews 45: 525–567. https://doi.org/10.1111/j.1469-185X.1970.tb01176.x

[B75] Parker GA (1984) Sperm Competition and the Evolution of Animal Mating Strategies. In: Smith RL (Ed.) , Sperm Competition and the Evolution of Animal Mating Systems. Academic Press, London, 1–60. https://doi.org/10.1016/B978-0-12-652570-0.50008-7

[B76] Parsons MJ (1983) Notes on the Courtship of *Troides oblongmaculatus* papuensis (Papilionidae) in Papua New Guinea. Journal of the Lepidopterists’ Society 37: 83–85.

[B77] Penz CM Francini RB (1996) New species of *Actinote* Hübner (Nymphalidae: Acraeinae) from southeastern Brazil. Journal of the Lepidopterists’ Society 50: 309–320.

[B78] Pierre J (1985a) Le sphragis chez les Acraeinae (Lepidoptera: Nymphalidae). Annales de la Société Entomologique de France 21: 393–398.

[B79] Pierre J (1985b) Systématique évolutive et spéciation chez les Lépidoptères du genre *Acraea* (Nymphalidae). I - Introduction et complexes ultraspécifiques. Annales de la Société entomologique de France 21: 5–27. Available from: http://gallica.bnf.fr/ark:/12148/bpt6k6129393g/f14.

[B80] Pierre J (1988) Les *Acraea* du Super-groupe «Egina» Révision et Phylogénie (Lepidoptera: Nymphalidae). Annales de la Société Entomologique de France 24: 263–287.

[B81] Pierre J (1992a) Systématique évolutive et cladistique: approche morphologique, spéciation et génation, application chez les *Acraea* (Lepidoptera, Nymphalidae). Bulletin de la Societé entomologique de France 97: 105–118.

[B82] Pierre J (1992b) Une nouvelle espèce d’Acraea (Lepidoptera Nymphalidae). Lambillionea 92: 308–310.

[B83] Pierre J Bernaud D (2009) Butterflies of the World 31: Nymphalidae 16: *Acraea*, subgenus Actinote. Goecke & Evers, Keltern, 5 pp.

[B84] Pierre J Bernaud D (2013) Butterflies of the World 39: *Acraea*, subgenus Acraea. Goecke & Evers, Keltern, 8 pp.

[B85] Pierre J Bernaud D (2014a) Butterflies of the World, Supplément 24, Le genre *Acraea* Fabricius, 1807: Liste systématique, synonymique et liste des noms infrasubspécifiques. Erich Bauer & Thomas Frankenbach, Goecke & Evers, Keltern, 30 pp.

[B86] Pierre J Bernaud D (2014b) Le genre *Acraea* Fabricius, 1807: Liste systématique, synonymique et liste des noms infrasubspécifiques. In: Bauer E Frankenbach T (Eds) Butterflies of the World, Supplement 24. Goecke & Evers, Keltern, 1–30.

[B87] Pliske TE (1975) Courtship Behavior of the Monarch Butterfly, *Danaus plexippus* L. Annals of the Entomological Society of America 68: 143–151. https://doi.org/10.1093/aesa/68.1.143

[B88] van der Poorten GM van der Poorten NE (2016) The Butterfly Fauna of Sri Lanka. Lepodon Books, Toronto, 418 pp.

[B89] Rawlins JE (1992) Life History and Systematics of the West Andean Moth *Aucula franclemonti* with Description of a New Species from Ecuador (Lepidoptera: Noctuidae: Agaristinae). Journal of the New York Entomological Society 100: 286–310.

[B90] Riley ND (1939) A new species of the genus *Armandia* (Lep. Papilionidae). Entomologist 72: 207–208.

[B91] Saigusa T (1973) A phylogeny of the genus *Luehdorfia*. Konchû-to-Shizen 8: 5–18.

[B92] Saigusa T Lee C (1982) A rare papilionid butterfly *Bhutanitis mansfieldi* (Riley): Its rediscovery new subspecies and phylogenetic position. Tyo to Ga 33: 1–24.

[B93] Scoble MJ (1992) The Lepidoptera: Form, Function and Diversity. Oxford University Press, Oxford, 420 pp.

[B94] Scott JA (1972) Mating of butterflies. Journal of Research on the Lepidoptera 11: 99–127.

[B95] Shuker DM Simmons LW (2014) The evolution of insect mating systems. Oxford University Press, Oxford. https://doi.org/10.1086/681480

[B96] Silberglied RE Shepherd JG Dickinson J Lou (1984) Eunuchs: The Role of Apyrene Sperm in Lepidoptera? The American Naturalist 123: 255–265. https://doi.org/10.1086/284200

[B97] Simmons LW (2001) Sperm Competition and Its Evolutionary Consequences in the Insects. Princeton University Press, Princeton, 456 pp.

[B98] Simonsen TJ de Jong R Heikkilä M Kaila L (2012) Butterfly morphology in a molecular age - Does it still matter in butterfly systematics? Arthropod Structure and Development 41: 307–322. https://doi.org/10.1016/j.asd.2012.04.006 10.1016/j.asd.2012.04.006 22583793

[B99] Solensky MJ Oberhauser KS (2009) Sperm precedence in monarch butterflies (*Danaus plexippus*). Behavioral Ecology 20: 328–334. https://doi.org/10.1093/beheco/arp003

[B100] van Son G (1963) The butterflies of Southern Africa: Part III Nymphalidae: Acraeinae. In: Transvaal Museum Memoir. Pretoria, 130 pp. [XXIX Plates.]

[B101] Sonnenschein M Hauser CL (1990) Presence of only eupyrene spermatozoa in adult males of the genus *Micropterix* Hübner and its phylogenetic significance (Lepidoptera: Zeugloptera, Micropterigidae). International Journal of Insect Morphology and Embryology 19: 269–276. https://doi.org/10.1016/0020-7322(90)90012-E

[B102] Sourakov A Emmel TC (1997) Mating habits in the genus *Acraea*, with a possible explanation for monosexual populations (Lepidoptera: Nymphalidae: Acraeinae). Tropical Lepidoptera 8: 33–35.

[B103] Takakura T (1967) Taiyo-no-ko usubashiro-cho no seikatsu (Life of the child the sun Parnassius glacialis). In: Iwase T (Ed.) Nihon-Konchuki . II. Cho no seikatsu. Kodansha, Tokyo, 75–106.

[B104] Thornhill R Alcock J (1983) The Evolution of Insect Mating Systems. Harvard University Press, Cambridge, 547 pp https://doi.org/10.4159/harvard.9780674433960

[B105] Timmermeyer N Gerlach T Guempel C Knoche J Pfann JF Schliessmann D Michiels NK (2010) The function of copulatory plugs in *Caenorhabditis remanei*: hints for female benefits. Frontiers in zoology 7. https://doi.org/10.1186/1742-9994-7-28 10.1186/1742-9994-7-28 PMC2987753 21044286

[B106] Tykac J (1951) Sphragis či sphragidoid u motýlů. Sphragis ou sphragidoid chez les Lépidoptères. Acta Societatis Entomologicae Cechosloveniae 48: 94–98.

[B107] Tyler HA Brown Jr KS Wilson KH (1994) Swallowtail Butterflies of the Americas, a study in biological dynamics, ecological diversity, biosystematics and conservation. Scientific Publishers, Gainesville, 375 pp.

[B108] Uhl G Nessler SH Schneider JM (2010) Securing paternity in spiders? A review on occurrence and effects of mating plugs and male genital mutilation. Genetica 138: 75–104. https://doi.org/10.1007/s10709-009-9388-5 19705286 10.1007/s10709-009-9388-5

[B109] Vlasanek P Konvicka M (2009) Sphragis in *Parnassius mnemosyne* (Lepidoptera: Papilionidae): male-derived insemination plugs loose efficiency with progress of female flight. Biologia 64: 1206–1211. https://doi.org/10.2478/s11756-009-0207-3

[B110] Walker WF (1980) Sperm utilization strategies in nonsocial insects. The American Naturalist 115: 780–799. https://doi.org/10.1086/283600

[B111] Waterhouse GA Lyell G (1914) The butterflies of Australia. A monograph of the Australian Rhophalocera. Angus and Robertson, Sydney, 239 pp.

[B112] Wedell N (2005) Female receptivity in butterflies and moths. Journal of Experimental Biology 208: 3433–3440. https://doi.org/10.1242/jeb.01774 16155216 10.1242/jeb.01774

